# Process Simulation and Cost Evaluation of Carbon Membranes for CO_2_ Removal from High-Pressure Natural Gas

**DOI:** 10.3390/membranes8040118

**Published:** 2018-11-30

**Authors:** Yunhan Chu, Xuezhong He

**Affiliations:** Department of Chemical Engineering, Norwegian University of Science and Technology, NO 7491 Trondheim, Norway; yunhan.chu@ntnu.no

**Keywords:** natural gas, carbon membranes, CO_2_ removal, process simulation, cost estimation, methane loss

## Abstract

Natural gas sweetening is required to remove the acid gas CO_2_ to meet gas grid specifications. Membrane technology has a great potential in this application compared to the state-of-the-art amine absorption technology. Carbon membranes are of particular interest due to their high CO_2_/CH_4_ selectivity of over 100. In order to document the advantages of carbon membranes for natural gas (NG) sweetening, HYSYS simulation and cost evaluation were conducted in this work. A two-stage carbon membrane process with recycling in the second stage was found to be technically feasible to achieve >98% CH_4_ with <2% CH_4_ loss. The specific natural gas processing cost of 1.122 × 10^−2^ $/m^3^ sweet NG was estimated at a feed pressure of 90 bar, which was significantly dependent on the capital-related cost. Future work on improving carbon membrane performance is required to increase the competitiveness of carbon membranes for natural gas sweetening.

## 1. Introduction

Natural gas (NG) is becoming one of the most attractive and growing fuels for world primary energy consumption because it is a cleaner energy source compared to other fossil fuels like coal and crude oil [1,2]. However, raw natural gas produced from gas wells usually contains light and heavy hydrocarbons (HHCs) and other impurities, such as H_2_O, H_2_S, and CO_2_. Natural gas sweetening is mandatory to remove the acid gases H_2_S and CO_2_ to meet the legal requirements and gas grid specifications. Various technologies, such as chemical absorption [3], pressure swing adsorption (PSA) [4,5,6], and membranes [7,8], have been used for CO_2_ removal from natural gas. Among them, conventional amine absorption is implemented in industrial processes and is still considered as the state-of-the-art technology for this application. However, membrane systems possess many advantages, such as small footprint, low capital and operating costs, environmental friendliness, and process flexibility [9], which show great potential for natural gas sweetening. However, commercial polymeric membranes used for natural gas sweetening, such as cellulose acetate (CA), cellulose triacetate (CTA), and polyimide (PI), have relatively low separation performance (i.e., low CO_2_/CH_4_ selectivity and low CO_2_ permeance) due to membrane compaction and plasticization [10], which lead to high costs due to a larger required membrane area and a shorter lifetime. This indicates the need to develop novel, high performance membranes materials. Recently, carbon nanotubes (CNTs)-reinforced polyvinyl amine (PVAm)/polyvinyl alcohol (PVA) in a blended fixed-site-carrier (FSC) membranes were reported to show good separation performance at moderate pressure operation (up to 40 bar) and relatively good long-term durability when exposed to different impurities [10]. However, there are still challenges to maintain separation performance at higher pressure of >40 bar (especially >80 bar in most natural gas plants). Mechanically strong carbon membranes can potentially address this challenge of being able to operate at high pressure without significant loss of separation performance. Different polymeric precursors, such as polyimides (PI) and cellulose derivatives, have been used for fabrication of carbon membranes [11,12,13,14,15]. Hollow fiber carbon membranes made from cellulose acetate showed high CO_2_/CH_4_ selectivity (>100) but only a moderate CO_2_ permeance (<0.1 m^3^(STP)/(m^2^·h·bar)) due to a thick wall with a symmetric structure at low pressure of <8 bar [16]. A recent study reported good separation performance at moderate and high pressure of up to 20 bar with a feed gas of 40% CO_2_/60% CH_4_ [17]. It should be noted that carbon membrane selectivity is usually much higher compared to commercial polymeric membranes (<30). High selectivity can significantly reduce operating cost and methane loss. However, a relatively lower CO_2_ permeance of carbon membrane will increase membrane unit cost due to a larger required membrane area. A membrane material with high performance of both CO_2_/CH_4_ selectivity and CO_2_ permeance can provide a competitive technology for CO_2_ removal from high-pressure natural gas. It should be noted that technology feasibility analysis should be conducted before bringing any membrane material into pilot demonstration and/or commercial application. Process design is essential for an energy-efficient membrane technology for natural gas sweetening, which usually depends on membrane separation performance, process operating parameters, such as feed CO_2_ concentration, as well as the product requirements, e.g., CH_4_ purity and CH_4_ loss. Several researches have reported on different polymeric membranes for CO_2_ removal from natural gas based on process design and simulation [18,19,20,21,22,23]. The latest two-stage CNT-reinforced FSC membrane systems for CO_2_ removal from moderate-pressure (40 bar) natural gas were reported by He et al. [24], and a lower natural gas processing cost was obtained compared to amine absorption. However, these membranes may not be competitive for high-pressure processes (e.g., >60 bar). Therefore, in this work, a techno-economic feasibility analysis was conducted to evaluate the advantages of carbon membranes for CO_2_ removal from natural gas. Moreover, attention was particularly paid to the influence of permeate pressure on membrane system performance, which has not been reported in the literature as yet.

## 2. Methods

### 2.1. Process Design

**Figure 1:**
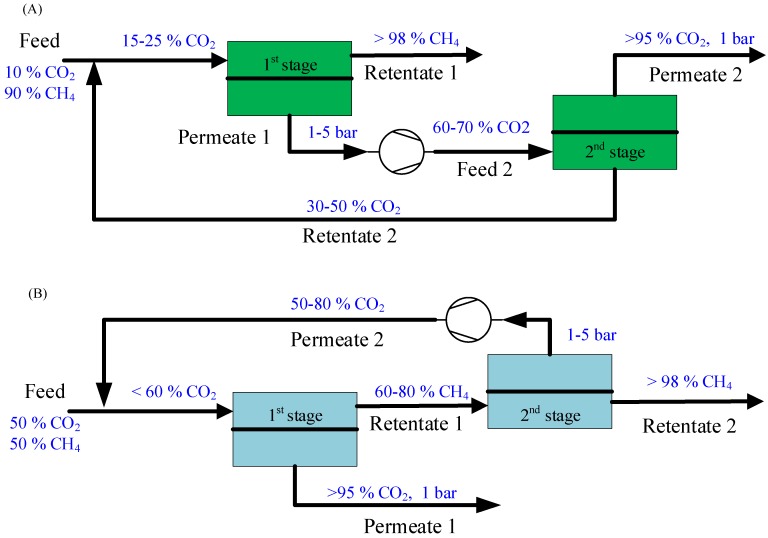
Membrane processes for natural gas (NG) sweetening, (**A**) 10% CO_2_ feed; (**B**) 50% CO_2_ feed.

Large conventional gas fields are becoming less accessible, which has leads to the need for embracing more challenging gas resources containing high CO_2_ and H_2_S [24]. CO_2_ content in natural gas is very much dependent on the fields and will usually increase as time passes. Natural gas with higher CO_2_ content are more challenging to process with conventional amine absorption due to the requirement of larger columns and higher energy consumption. However, membrane systems show a great flexibility to tolerate the variations in feed CO_2_ content, which is particularly relevant for enhanced gas recovery where CO_2_ content in the produced natural gas changes over time. Moreover, membrane technology would be beneficial for offshore platform operations due to its small footprint and low energy consumption. Process design is essential to provide an energy-efficient membrane technology for natural gas sweetening. Two-stage membrane systems have been previously reported for CO_2_ removal from natural gas using FSC membranes [21,24], and recycling in the second stage is required to achieve a low methane loss. Therefore, two membrane processes, illustrated in Figure 1, are required for natural gas sweetening with different feed CO_2_ content to achieve the separation requirements. Sweet natural gas with high CH_4_ purity (>98 vol.%) is produced in the first-stage retentate stream for natural gas with a low CO_2_ content (e.g., 10 vol.%), as shown in Figure 1A. Recycling of retentate stream in the second stage can achieve a low CH_4_ loss (<2%). For natural gas with high CO_2_ content (e.g., 50 vol.%), high-purity sweet natural gas can only be produced in the second-stage retentate stream, as indicated in Figure 1B. The recycling of the second-stage permeate can secure a low methane loss.

### 2.2. Simulation Basis

**Figure 2:**
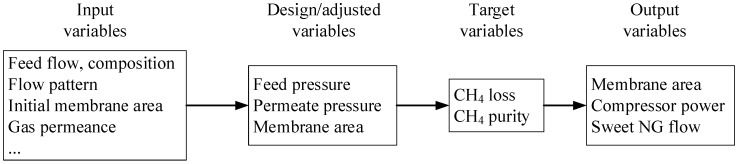
Illustration of simulation workflow of membrane systems for CO_2_ removal from natural gas.

The performance of carbon membranes reported in a previous work [17] was used as the simulation input in this study. It is worth noting that gas permeance and selectivity are pressure-dependent, as described in Table 1 (membrane performance at different pressure was predicted based on the model fitted to the experimental data), which provides a more accurate evaluation on technology feasibility. Two simulation scenarios with various feed composition, feed pressure (p_F_), and permeate pressure (p_P_), listed in Table 2, were conducted by HYSYS integrated with ChemBrane (in-house membrane module [25]). The detailed membrane model has been described in a previous work [17]. The simulation workflow of hollow fiber carbon membrane systems for CO_2_ removal from natural gas in HYSYS is illustrated in Figure 2, which also outlines the input variables (e.g., flow pattern, initial membrane area, feed flow, etc.). The design variables—feed and permeate pressures—were investigated to document their influence on gas processing cost. Membrane area was adjusted to achieve the target variables, i.e., CH_4_ purity and CH_4_ loss in the first and second stage, respectively. The output variables—required membrane area, compressor power demand, and sweet NG flow—were used for cost evaluation. High feed pressures of 50–90 bar were investigated in the process simulations. Two-stage carbon membrane systems were designed to evaluate the technology feasibility of carbon membrane systems for CO_2_ removal from a 50,000 m^3^(STP)/h natural gas plant (with 10 and 50 vol.% CO_2_ in feed) at 30 °C. The CH_4_ purity of >98 vol.% and CH_4_ loss of <2% were defined as the separation targets.

### 2.3. Cost Evaluation

The cost of the major equipment (e.g., compressor and membrane unit) was estimated by the cost model reported by He et al. [17]. The project time was set to 20 years, and the purchase cost of axial compressor (450–3000 kW) at base condition was estimated based on the CAPCOST 2012 as follows [26]:

(1) $$\log_{10}C_{p}^{0}=2.2891+1.3604\log_{10}\left(Q\right)-0.1027{\left[\log_{10}\left(Q\right)\right]}^{2}$$

where *Q* is compressor capacity (kW). Compressors with nickel materials are considered for high-pressure operation, and the bare module factor of 15.9 was used. The chemical engineering plant cost index (CEPCI) for the equipment of 567.5 in 2017 was used to adopt all inflation adjustments (397 in 2012). Therefore, the total capital cost (*C_TM_*) of rotary compressors, including the contingency and contractor fee in addition to the direct and indirect cost (a factor of 1.18), was calculated by Equation (2):

(2) $$C_{TM}=1.18\times 15.9\times \frac{567.5}{397}\times \overset{n}{\underset{i=1}{\sum }}C_{p,i}^{0}$$

where *n* is the total number of individual compressor units. The membrane cost of $50–100 per m^2^ was applied to estimate carbon membrane skid cost (*C_M_*), which included the membrane installation cost. Moreover, the membrane lifetime was set to 5 years [17,27]. The annual capital-related cost (*CRC*) was estimated by Equation (3):

(3) $$CRC=0.2\times \left(C_{TM}+C_{M}\right)$$

For annual operating expenditure (*OPEX*), only electricity cost was considered in order to simplify cost estimation (0.04 $/kWh). Assuming the operation time of 8000 h per year, the annual *OPEX* can be estimated as follows:

(4) OPEX=0.04×Q×8000

The specific natural gas processing cost (*C^S^*, $/m^3^ sweet *NG*) can then be estimated according to Equation (5):

(5) $$C^{S}=\frac{CRC+OPEX}{Annual\;total\;NG\;production}$$

*C^S^* was employed to evaluate economic feasibility of carbon membrane system for CO_2_ removal from high-pressure natural gas.

## 3. Results and Discussion

The major equipment—the compressors and membrane units—were designed and operated in a particular way for CO_2_ removal from high-pressure natural gas to meet the specific requirements given in Table 1. The designed two-stage membrane systems shown in Figure 1 were used for the simulation of different scenarios listed in Table 2. The influence of the operating parameters—feed CO_2_ concentration, feed pressure, and permeate pressure—in the second stage on membrane system performance were investigated. Cost minimization was also performed to identify the optimal operational condition in a specific membrane process.

### 3.1. Feed Pressure Influence with 10% CO_2_ Feed

The feed gas pressure was varied from 50–90 bar in Scenario 1 to investigate its influence on power demand and required membrane area. Table 3 shows the dependence of the power demand and the total carbon membrane area on feed pressure. As can be seen, the power demand of the compressors increased with the increase in feed pressure, while the required membrane area decreased due to a higher driving force for gas transport through membranes. A cost estimation based on Equation (5) was conducted to identify the optimal feed pressure (membrane cost of 100 $/m^2^ was used), and the results are given in Table 3. The lowest natural gas processing cost of 1.122 × 10^−2^ $/m^3^ sweet NG was found at the feed pressure of 90 bar. Although this cost is higher compared to an amine absorption system of 6.4 × 10^−3^ $/Nm^3^ reported by Peters et al. [21], increasing carbon membrane performance can potentially bring down the total cost of the membrane system. It should be noted that natural gas feed pressure is dependent on the gas wells and the required pretreatment units, and the gas plant with higher pressure sour natural gas requires lower CO_2_ removal cost due to a higher driving force in the first stage without extra energy cost.

### 3.2. Permeate Pressure Influence with 50% CO_2_ Feed

**Figure 3:**
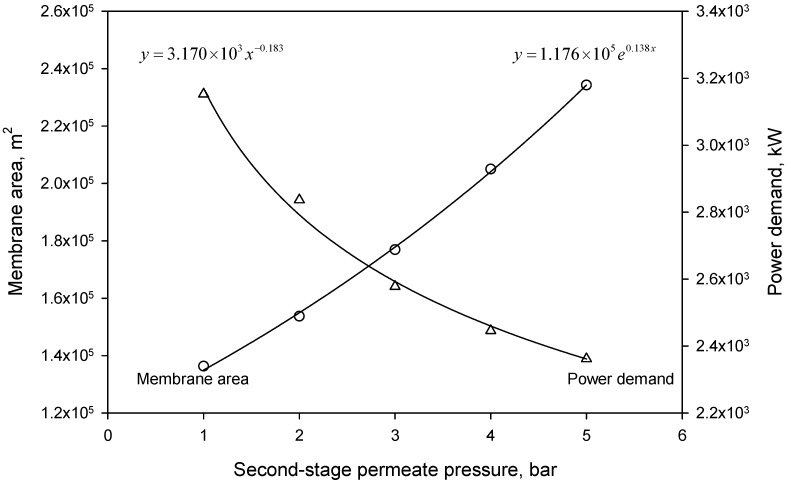
Dependence of required membrane area and power demand on the second-stage permeate pressure.

The influence of the second-stage permeate pressure on the membrane system performance was conducted on Scenario 2, where a natural gas with 50% CO_2_ content was fed into the system at 50 bar. The first-stage permeate pressure was set to 1 bar for high-purity CO_2_ production, while the second-stage permeate pressure varied from 1–5 bar. The simulation results are shown in Figure 3. Increasing the second-stage permeate pressure resulted in a decrease in the power demand of the compressors due to a higher inlet pressure, and the dependence was found to be as follows:

(6) $$Q=3.170\times 10^{3}p_{P}^{-0.183}$$

Meanwhile, the required membrane area increased with the increase in permeate pressure, as expressed in Equation (7):

(7) $$A=1.176\times 10^{5}e^{0.138p_{P}}$$

**Figure 4:**
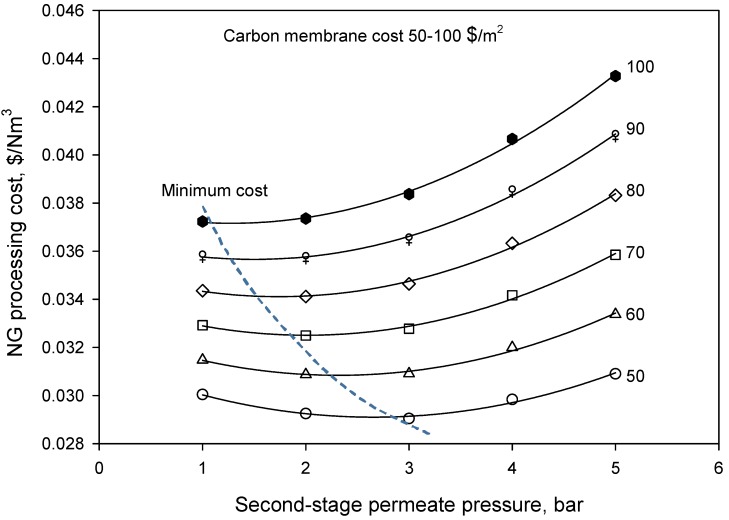
The influence of the second-stage permeate pressure on the NG processing cost.

In order to identify the optimal permeate pressure in the second stage, cost estimation was performed based on Equation (5), and the results are shown in Figure 4. It was found that the minimum NG processing costs at different second-stage permeate pressures were dependent on the membrane cost. Compared to the FSC membranes for natural gas sweetening reported in a previous work [24], the investigated carbon membrane system presented a higher NG processing cost, as listed in Table 4. However, it should be noted that a much higher pressure of 50 bar was simulated for the carbon membranes compared to the FSC membrane system of 20 bar. Moreover, fixed gas permeance was used for the FSC membranes at difference pressures, which most likely does not exist in a real system. In addition, the specific required membrane area for the carbon membranes was found to be much larger compared to the FSC membranes due to a much lower gas permeance of the carbon membranes. Therefore, future work on improving gas permeance of carbon membrane is significantly required to increase its competitiveness for natural gas sweetening.

### 3.3. Sensitivity Analysis of Membrane Performance

**Figure 5:**
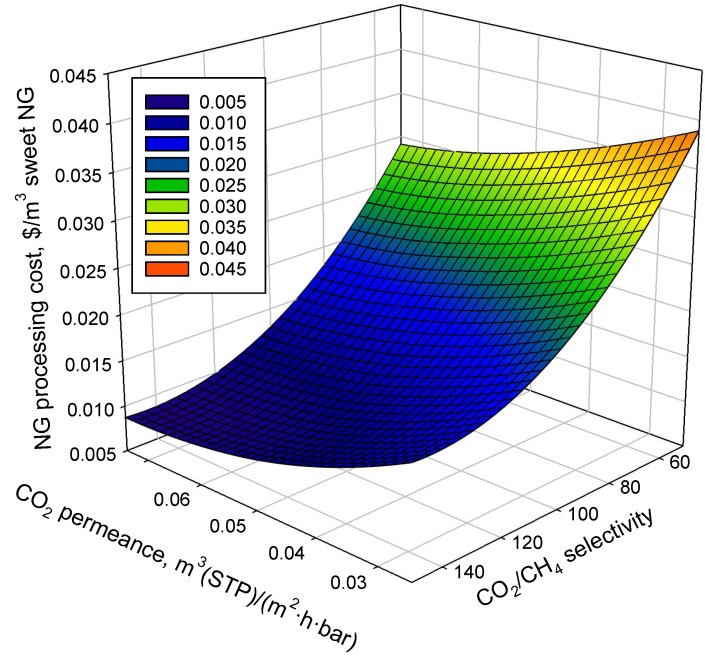
The influence of membrane separation performance on NG processing cost.

The sensitivity analysis of both CO_2_ permeance and CO_2_/CH_4_ selectivity (up to three times the experimental data) on the natural gas processing cost were investigated. The process simulations were conducted based on Scenario 2 with a permeate pressure of 1 bar, as shown in Table 2. The influence of membrane performance on the NG processing cost is shown in Figure 5. It can be seen that the increase in both CO_2_ permeance and CO_2_/CH_4_ selectivity could reduce the specific cost; selectivity had a more significant effect as it dramatically reduced energy consumption. Thus, future research direction should be focused on the improvement of CO_2_/CH_4_ selectivity of carbon membranes at high-pressure operations, which is crucial for the processing of natural gas with high CO_2_ content.

## 4. Conclusions

In this study, the designed two-stage carbon membrane system with recycling in the second stage was shown to produce high-purity CH_4_ (>98 vol.%) with a low CH_4_ loss of <2% based on HYSYS simulation. The specific natural gas processing cost was found to be significantly dependent on the capital-related cost, which could be brought down by reducing the membrane skid cost. Moreover, the second-stage permeate pressure had significant influence on the cost for processing natural gas with high CO_2_ content. The carbon membrane performance, especially CO_2_/CH_4_ selectivity, was also found to have a great effect on the natural gas processing cost. Further improvements in carbon membrane performance can potentially increase its competitiveness for this application.

## Figures and Tables

**Table 1:** The simulation basis for natural gas sweetening using carbon membrane system.

Parameters	Values
Feed flow, m^3^(STP)/h	50,000
Feed gas composition	Table 2
Feed/permeate pressure, bar	Table 2
Feed temperature, °C	30
CO_2_ permeance, m^3^(STP)/(m^2^·h·bar) *	$P_{{\mathrm{CO}}_{2}}=0.3137\times P^{-0.65}$
CO_2_/CH_4_ selectivity *	$S_{{\mathrm{CO}}_{2}/{\mathrm{CH}}_{4}}=176\times P^{-0.323}$
CH_4_ purity, vol.%	>98
CH_4_ loss, %	<2
Membrane area, m^2^	Adjusted

* tested at feed pressure below 20 bar [17].

**Table 2:** List of different simulation scenarios.

Scenario	Feed Gas Composition, vol.%		Feed Pressure (p_F_), bar	Permeate Pressure (p_P_), bar	
	CO_2_	Methane		First Stage	Second Stage
Case 1	10	90	50–90	1	1
Case 2	50	50	50	1	1–5

**Table 3:** The simulation and cost estimation results of Scenario 1.

Feed Pressure, Bar	Membrane Area, m^2^	Power Demand, kW	CRC, $	OPEX, $	*C^S^*, $/m^3^ Sweet NG
50	1.19 × 10^5^	1109	4.00 × 10^6^	3.55 × 10^5^	1.278 × 10^−2^
60	1.06 × 10^5^	1154	3.78 × 10^6^	3.69 × 10^5^	1.219 × 10^−2^
70	9.46 × 10^4^	1180	3.58 × 10^6^	3.78 × 10^5^	1.162 × 10^−2^
80	8.94 × 10^4^	1238	3.54 × 10^6^	3.96 × 10^5^	1.156 × 10^−2^
90	8.27 × 10^4^	1256	3.42 × 10^6^	4.02 × 10^5^	1.122 × 10^−2^

**Table 4:** Comparisons between carbon membranes and fixed-site-carrier (FSC) membranes for CO_2_ removal from natural gas.

Parameters	Carbon Membrane in this Work	FSC Membranes [24]
Feed pressure, bar	50	20
Second-stage permeate pressure, bar	1–5	1
CH_4_ purity in sweet NG, vol.%	98	96.08
CH_4_ loss, %	2	0.35
Specific power consumption, kWh/Nm^3^ sweet NG	0.1	2.43 × 10^−2^
Specific membrane area, m^2^/Nm^3^ sweet NG	9.90	0.56
*C^S^*, $/Nm^3^ sweet NG	4.33 × 10^−2^ *	4.22 × 10^−3^

* based on a carbon membrane cost of 100 $/m^2^.
